# Alternative approach of hepatocellular carcinoma surveillance: abbreviated MRI

**DOI:** 10.20517/2394-5079.2020.50

**Published:** 2020-09-01

**Authors:** Ryan L. Brunsing, Kathryn J. Fowler, Takeshi Yokoo, Guilherme Moura Cunha, Claude B. Sirlin, Robert M. Marks

**Affiliations:** 1 Department of Radiology, Stanford University, Stanford, CA 94305, USA; 2 Liver Imaging Group, Department of Radiology, University of California San Diego, San Diego, CA 92093, USA; 3 Department of Radiology, University of Texas Southwestern Medical Center, Dallas, TX 75390, USA; 4 Department of Radiology, Naval Medical Center San Diego, San Diego, CA 92134, USA; 5 Department of Radiology, Uniformed Services University of the Health Sciences, Bethesda, MD 20892, USA

**Keywords:** Abbreviated magnetic resonance imaging, cirrhosis, Hepatitis B, hepatocellular carcinoma, surveillance, magnetic resonance imaging

## Abstract

This review focuses on emerging abbreviated magnetic resonance imaging (AMRI) surveillance of patients with chronic liver disease for hepatocellular carcinoma (HCC). This surveillance strategy has been proposed as a high-sensitivity alternative to ultrasound for identification of patients with early-stage HCC, particularly in patients with cirrhosis or obesity, in whom sonographic visualization of small tumors may be compromised. Three general AMRI approaches have been developed and studied in the literature - non-contrast AMRI, dynamic contrast-enhanced AMRI, and hepatobiliary phase contrast-enhanced AMRI - each comprising a small number of selected sequences specifically tailored for HCC detection. The rationale, general technique, advantages and disadvantages, and diagnostic performance of each AMRI approach is explained. Additionally, current gaps in knowledge and future directions are discussed. Based on emerging evidence, we cautiously recommend the use of AMRI for HCC surveillance in situations where ultrasound is compromised.

## INTRODUCTION

Imaging-based surveillance for hepatocellular carcinoma (HCC) aims to detect early-stage, potentially curable tumors in asymptomatic high-risk patients to prolong life. First introduced about four decades ago, it is now an established part of routine clinical care for patients with chronic hepatitis B or cirrhosis in many countries across the globe. A randomized controlled trial of over 18,000 people with active or chronic hepatitis B showed that semi-annual screening with a combination of ultrasound (US) and serum alpha fetoprotein reduced HCC-related mortality by 37%^[1]^. Based on the above findings, other studies^[2,3]^, cost and availability considerations, US is recommended by most national and international hepatology societies for HCC surveillance^[4–10]^. Since surveillance US does not permit a definitive diagnosis of HCC, positive surveillance US exams prompt additional diagnostic tests, usually a contrast-enhanced multiphase computed tomography (CT) or magnetic resonance imaging (MRI). Patients with negative US exams return for routine surveillance US examinations, usually at six-month intervals.

Despite universal recommendation for use of US in HCC surveillance, the efficacy of this modality is disappointing. US has low sensitivity for HCC^[11,12]^, in particular for patients with early-stage tumors^[12–14]^, ascites, cirrhosis or obesity^[15–17]^. Meta-analyses indicate that the sensitivity of surveillance US to detect small (e.g., ≤ 2 cm) HCCs in patients with cirrhosis is less than 50%, i.e., more than half of patients with potentially curable cancers are missed and may progress to advanced, incurable disease before diagnosis^[14,18,19]^. Delayed diagnosis defeats the purpose of surveillance, which aims to detect patients with very early- or early-stage HCC^[20]^, allowing for curative therapies^[21]^. The failure to detect early disease contributes to HCC-related mortality^[22]^.

A more sensitive surveillance test might improve outcomes in patients at risk for HCC. Compared to US, both CT and MRI have superior reported diagnostic sensitivity to identify patients with HCC^[16,19^, including those with early-stage tumors^[15]^, however they also pose challenges as surveillance tools. CT requires injection of iodinated intravenous contrast agents, which can cause allergic reactions and possibly nephrotoxicity, potentially limiting the use of this modality in certain populations. In addition, CT exposes patients to ionizing radiation, an important consideration in younger or middle-aged adults with well-compensated cirrhosis. Conventional MRI provides higher sensitivity than CT^[16,19]^, but also requires administration of intravenous contrast material; moreover, long exam duration, interpretation complexity, and high cost hinder its suitability for surveillance.

Motivated to provide higher sensitivity than US while avoiding the limitations of CT and conventional MRI, investigators have developed abbreviated MRI (AMRI) protocols that rely on a small number of select sequences specifically tailored for HCC detection^[12,23–36]^. The rationale is that reduced scanner time decreases costs and complexity, while improving patient comfort, without significantly compromising HCC detection. AMRI also simplifies workflow and possibly interpretation, while utilizing fewer resources. Recent studies suggest that AMRI might be a high-sensitivity and feasible alternative to US for HCC surveillance, and a recent Markov model-based cost-utility analysis suggested AMRI-based HCC-surveillance may be the most cost-effective strategy^[37]^.

The purpose of this article is to review emerging concepts on AMRI-based HCC surveillance, including technical aspects, diagnostic performance, current gaps in knowledge, and future directions.

## AMRI: APPROACHES

**Figure 1.**
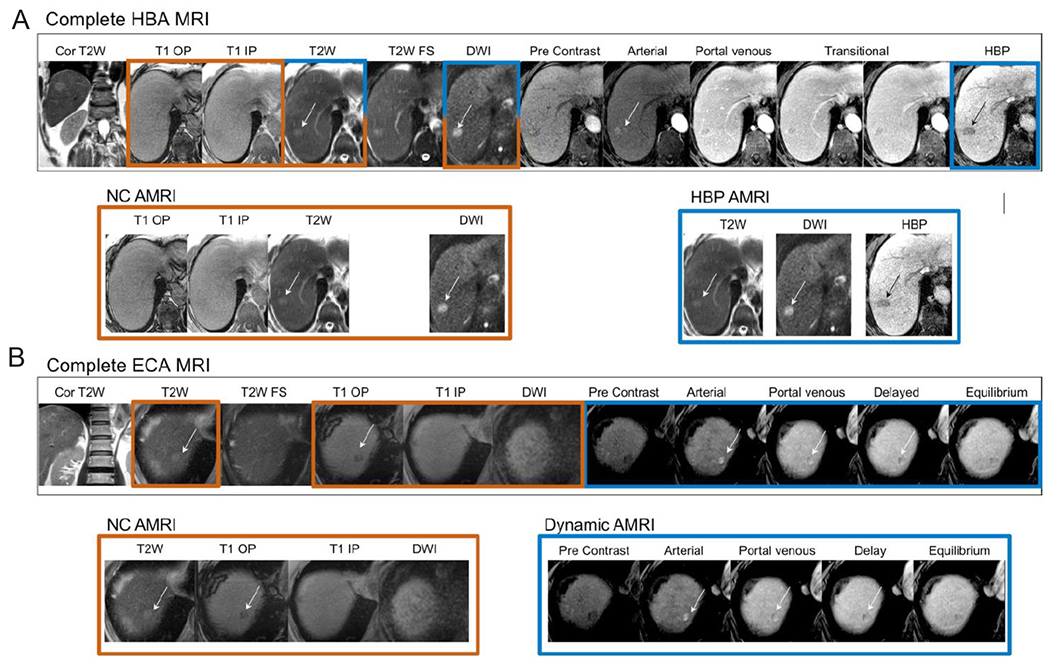
Complete MRI exams (A: HBA MRI; B: ECA) disaggregated into each of the three AMRI approaches (NC-AMRI, HBP AMRI and Dynamic AMRI). MRI: magnetic resonance imaging; NC AMRI: non-contrast abbreviated MRI; HBA: hepatobiliary agents; ECA: extracellular contrast agents; HBP: hepatobiliary phase

Three general AMRI approaches have been developed: non-contrast AMRI, dynamic contrast-enhanced AMRI, and hepatobiliary phase contrast-enhanced (HBP) AMRI. All can be completed in approximately 10 min or less of scanner time, considerably less than a complete or conventional MRI exam of the liver, which typically requires half an hour or more. Figure 1 illustrates how a complete MRI exam can be disaggregated into each of the three AMRI approaches. The approaches, discussed in detail below, are summarized in Table 1 along with their advantages and disadvantages.

## NON-CONTRAST AMRI

### Imaging

The simplest approach to MRI-based HCC surveillance is non-contrast abbreviated MRI (NC-AMRI), which implements up to three sequences without administering contrast material:

#### T1 weighted in-phase and out-of-phase imaging

**Figure 2.**
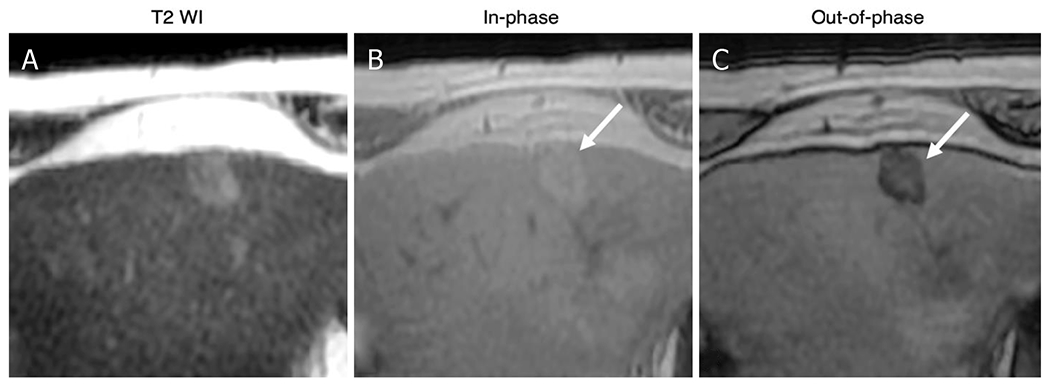
Intralesional fat: 80-year-old male with HCV cirrhosis. Images show a 18 mm observation in the left lobe. The lesion has ancillary features favoring HCC including mild hyperintense on T2WI (A) as well as intralesional fat in the mass more than adjacent liver. The latter is characterized by signal drop from In-phase (B) to Out-of-phase (C) images (arrows). HCV: hepatitis C virus; HCC: hepatocellular carcinoma

With current MRI systems, T1-weighted in-phase and out-of-phase images of the liver can be acquired in a single breath-hold. These images can detect HCC nodules that are either hypointense or hyperintense relative to liver, but they generally have low sensitivity for early-stage HCC, which is usually hypointense on this sequence. In-phase and out-of-phase (IP/OOP) images can also provide information on fat [Figure 2] or iron content, which might be useful for differentiating suspicious from benign lesions. In particular, nodules that differ in fat content from background liver (either more fat or less) based on IP/OOP signal characteristics signal characteristics or nodules with lower iron content than background liver (iron sparing) are suspicious for malignancy. By comparison, nodules with higher iron content (siderotic) are usually non-malignant; if only siderotic nodules are detected, the exam is considered negative for HCC.

#### T2 weighted imaging

The main purpose of including T2 weighted imaging is to help differentiate suspicious from benign lesions. Marked T2 hypointensity or marked T2 hyperintensity suggest that a lesion is non-malignant, whereas mild-to-moderately increased T2 signal, relative to the background liver parenchyma, is more concerning for HCC in high-risk patients^[38]^. T2-weighted imaging may also improve sensitivity by detecting T2-hyper intense HCC nodules that are difficult to see for various reasons on the other sequences; the incremental benefit is likely to be modest given the relatively low sensitivity of this sequence for small HCC nodules.

#### Diffusion weighted imaging

Inclusion of diffusion weighted imaging (DWI) increases sensitivity^[39–41]^ by detecting lesions based on restricted diffusion, which is thought to reflect hypercellularity. Some DWI features may also be used to help differentiate HCC from non-HCC malignancy, such as intrahepatic cholangiocarcinoma (iCCA), which often has a more targetoid appearance^[42,43]^. The highest b-values have ranged from 500-800 s/mm^2^ for NC-AMRI studies.

### Reporting

**Figure 3.**
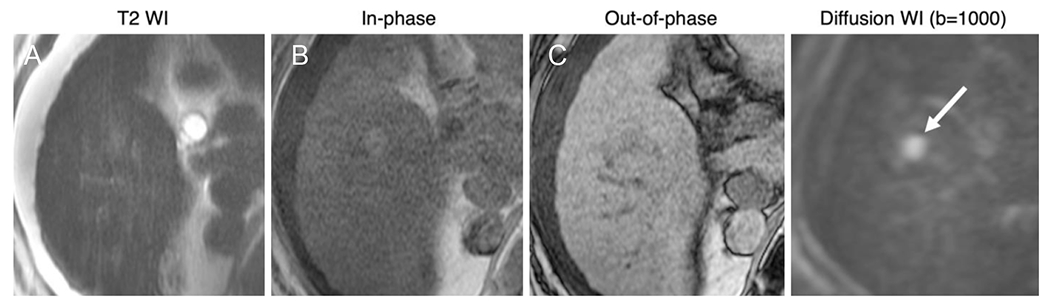
Positive NC-AMRI examinations: 66-year-old male with HCV cirrhosis. Images show a 14 mm observation in seen in the right lobe. While subtle on T2WI (A) and T1WI (B, C), the presence of restricted diffusion (arrow) favors malignancy. HCV: hepatitis C virus; NC AMRI: non-contrast abbreviated magnetic resonance imaging

NC-AMRI exams can be interpreted as positive in the setting of a focal observation meeting any of the above described criteria [Figure 3]. A positive examination would warrant a call back diagnostic study to provide a definitive diagnosis of HCC. Features that suggest non-HCC malignancy do not affect the need for call-back but might guide the radiologist’s choice of modality and contrast agent.

### Advantages

NC-AMRI offers several advantages. By avoiding gadolinium-based contrast agent (GBCA) administration, this approach curtails costs, avoids IV placement, saves time, and simplifies workflow. There is no need for image acquisition timing, and images compromised by respiratory or other motion artefacts can simply be repeated. It also eliminates any GBCA-associated risks, including rare but potentially serious adverse reactions^[31]^, theoretical concerns about gadolinium deposition in the brain^[44,45]^, and the remote possibility of nephrogenic systemic sclerosis, a disorder unique to patients with acute kidney injury or severely compromised renal function receiving high doses of certain GBCAs^[46]^.

### Disadvantages

The main disadvantage of NC-AMRI is that it relies exclusively on unenhanced images, which tend to have a relatively low contrast to noise ratio, potentially diminishing the visibility of HCC nodules as compared to post contrast sequences used in the other AMRI approaches. The inclusion of DWI, a high-contrast sequence, can aid in detecting liver lesions^[47]^, thereby improving sensitivity. However, DWI is technically challenging and often suffers from a variety of artifacts^[48]^ that can cause blind spots, most often near the liver dome or in the left lobe. Many early stage HCCs may not exhibit restricted diffusion relative to liver. In addition, HCC may be isointense to liver on T2 weighted imaging^[49]^ or obscured by altered signal in the liver parenchyma in the setting of cirrhosis. Such HCCs may be difficult to visualize on NC-AMRI.

### Studies to date

Several studies have retrospectively assessed the performance of a simulated NC-AMRI (derived by extracting only the non-contrasted sequences from a complete MRI), most utilizing all three sequences outlined above^[23,25,32]^, and some utilizing DWI alone^[34,36]^ [Table 2]. While these studies found favorable sensitivities ranging from 84%-92% on a per-patient basis, sensitivity was 78% on a per-lesion basis in one study that used liver explant pathology as the reference standard MC^[36]^. Most of these were retrospective studies in predominantly hepatitis-B population without advanced cirrhosis, enriched with a high prevalence of malignancy. Only one study thus far prospectively evaluated the performance of NC-AMRI in an HCC surveillance population^[34]^. Using DWI alone, this study demonstrated a sensitivity of 83% and sensitivity of 98%. However, a small number of incident HCCs (*n* = 6) and low prevalence of Child Pugh status B or C cirrhosis (< 6%) limit the generalizability of this result. To our knowledge, this study and a HBP-AMRI study discussed below^[31]^ are the only two studies to-date evaluating the performance of AMRI interpreted prospectively in the clinical setting.

### Summary statement

The strengths of NC-AMRI are maximum reduction in cost due to lack of contrast, minimum patient risk, simplified workflow, and the ability to repeat sequences compromised by motion or other resolvable artifacts. However, the generalizability of existing data, in particular to Western surveillance populations, is challenged by the enrichment of study populations with malignant lesions, preponderance of hepatitis B patients, and low prevalence of advanced (e.g., Child-Pugh B) cirrhosis. It is likely that the sensitivities and performance of NC-AMRI may be less favorable in North American or European populations due to differences in body habitus, etiologies of liver disease, and severity of cirrhosis. HCC detection accuracy for full NC-AMRI needs to be validated in prospective studies in surveillance patient cohorts. To this end, a randomized control trial has been initiated in a Korean population directly comparing NC-AMRI to US for HCC surveillance^[50]^, but similar studies will be needed in non-Asian populations before this approach can be widely recommended. Ultimately, the performance and clinical utility of this approach will be determined mainly by DWI, which provides higher lesion conspicuity than the other sequences, thus optimizing this sequence will be essential.

## DYNAMIC AMRI

### Imaging

Dynamic contrast-enhanced AMRI (Dynamic-AMRI), one of two AMRI strategies that utilize GBCAs, acquires dynamic contrast enhanced images using T1-weighted images with fat suppression following administration of an extracellular contrast agent. The dynamic component refers to images acquired at predetermined and successive phases to detect and characterize HCCs based on the vascular alterations of hepatocarcinogenesis. These phases include the following:

#### Pre-contrast imaging

The pre-contrast images provide a baseline from which all post-contrast images are assessed for contrast enhancement. Pre-contrast images also allow detection of intrinsic T1 hyperintense observations, and for confirming that any hyperintensity on post contrast images represents true contrast enhancement. With modern MRI systems, it is possible to collect IP/OOP images simultaneously with the pre-contrast T1-weighted images (i.e., no additional acquisition is needed). If such images are acquired, they may permit assessment of relative fat or iron content relative to liver, as described for NC-AMRI.

#### Arterial phase imaging

Arterial phase (AP) is the time point after contrast injection at which tumor enhancement via arterial inflow is expected to be maximal. This usually occurs when portal veins are moderately to fully enhanced but the hepatic veins are not yet enhanced by antegrade flow. Appropriate timing of the AP is essential and can be achieved with reasonable consistency using current bolus-tracking technology or other methods^[51]^. This sequence is used to assess arterial phase hyperenhancement (APHE), meaning enhancement greater than background liver parenchyma in the AP. Thought to reflect the arterialization of HCC during hepatocarcinogenesis, APHE is one of the defining imaging features of HCC and is required for imaging-based diagnosis in high-risk patients, per Liver Imaging Reporting and Data System (LI-RADS)^[9]^.

#### Portal venous phase imaging

Portal venous phase (PVP) is the time point after contrast injection at which the portal veins are fully enhanced and the hepatic veins are enhanced by antegrade flow^[9]^, occurring approximately 40 sec after AP when the liver is expected to be at its peak enhancement. Portal and hepatic vein anatomy and patency are assessed on this phase, including the presence of tumor in vein, which indicates macrovascular invasion. Washout appearance and enhancing capsule appearance, other defining imaging features of HCC, may be detected if present.

#### Delayed phase imaging

Delayed phase (DP) images are usually acquired 2-5 min after injection. Washout appearance and enhancing capsule appearance are usually most conspicuous on the DP images.

### Reporting

**Figure 4.**
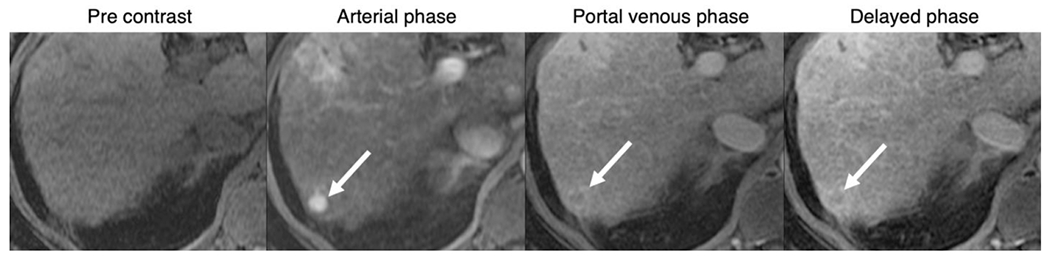
Positive dynamic-AMRI examination: 80-year-old male with HCV cirrhosis, images show a 11 mm observation in segment 7. The lesion has major features of HCC including nonrim APHE, washout and enhancing capsule (arrows) indicating definite HCC (LI-RADS-5). AMRI: abbreviated magnetic resonance imaging; HCV: hepatitis C virus; HCC: hepatocellular carcinoma; LI-RADS: Liver Imaging Reporting and Data System; APHE: arterial phase hyperenhancement

Reporting of dynamic-AMRI is based on the major features of HCC as defined by LI-RADS [Figure 4]. An exam detecting a mass, meeting criteria for HCC (i.e., LR-5), should be reported as a positive result. The reporting and follow-up recommendations for exams showing indeterminate lesions (i.e., LR-3 or LR-4) based on Dynamic-AMRI has not been standardized.

### Advantages

Dynamic-AMRI offers unique advantages. The defining imaging features of HCC (i.e., the LI-RADS major features of size, APHE, washout appearance, and enhancing capsule appearance) are determined from dynamic imaging. When a liver observation meets the required diagnostic criteria, dynamic AMRI alone suffices for definitive diagnosis of HCC per LI-RADS (i.e., LR-5). It also permits the diagnosis of tumor in vein (TIV). Additionally, it provides cost benefits, as the contrast agents used in dynamic AMRI are typically less expensive than the contrast agent (gadoxetate disodium) required for HBP-AMRI^[52]^. Some investigators have used coronal T2 imaging for localizer sequences, which can aid in characterizing benign lesions such as simple cysts and hemangiomas.

### Disadvantages

The disadvantages of dynamic-AMRI relate to the lack of additional non-contrast sequences, which may provide ancillary imaging features otherwise not available from the dynamic images^[53]^. The inability of dynamic-AMRI to evaluate these features may cause miscategorization of observations. In particular, dynamic-AMRI might over-categorize some vascular pseudolesions (e.g., arterio-portal shunts) as indeterminate (LR-3), potentially leading to unnecessarily close follow up. In theory, dynamic-AMRI also might under-categorize some early or small HCCs as LR-3, potentially delaying diagnosis, but the frequency with which this occurs is thought to be low. HCC detection by dynamic-AMRI depends on the timing and quality of arterial-phase imaging, which cannot be repeated if these images are mistimed or degraded by motion artifact or other problems. Finally, dynamic-AMRI requires a power injector for bolus intravenous administration of GBCA, which may not be available at all facilities and introduces complexity.

### Studies to date

A few studies to date have retrospectively assessed the performance of a simulated dynamic-AMRI (derived by extracting only the dynamic sequences from a complete MRI) for HCC detection in patients with cirrhosis [Table 2]. These studies have shown that dynamic AMRI is diagnostically similar to complete MRI for HCC detection^[26,27]^, with per-patient reported sensitivity and specificity of 94% and 88%, respectively^[26]^. However, these studies were conducted in diagnostic cohorts, in whom complete MRIs were indicated for known or clinically suspected liver lesions, which may have caused inflation in the sensitivity estimates. Dynamic-AMRI has yet to be tested prospectively in an HCC surveillance population.

### Summary Statement

Dynamic-AMRI can characterize the defining imaging features of HCC and allows the detection and diagnosis of HCCs in a single surveillance exam. The absence of T2 weighted and DWI sequences, however, may cause diagnostic uncertainty, particularly for benign vascular pseudolesions, and lead to unnecessary short interval follow-up or call-back. The requirements for a power injector and for precise arterial phase timing complicate the workflow compared to other AMRI approaches. HCC detection accuracy for dynamic AMRI needs to be validated prospectively in a surveillance patient cohort.

## HEPATOBILARY-PHASE AMRI

### Imaging

HBP contrast-enhanced AMRI (HBP-AMRI), the other AMRI approach that utilizes GBCA, is performed after administration of the hepatobiliary agent, gadoxetate disodium. The sequences include:

#### Hepatobiliary phase imaging

Acquired about 15-20 min following the administration of gadoxetate, when parenchymal enhancement with this agent is expected to be maximal, the hepatobiliary phase T1-weighted images provide high contrast-to-noise for lesion detection. In the hepatobiliary phase (HBP masses that are not of benign hepatocellular nature (e.g., HCCs and non-HCC malignant neoplasms) are hypointense relative to the high signal background liver, creating high liver to lesion contrast and increasing sensitivity. Hepatobiliary phase hypointensity is not specific for malignant nodules, however, and can be seen in benign non-hepatocellular entities, such as cysts and hemangiomas. Hence, any detected lesion must be correlated on T2-weighted imaging. If IP/OOP images are acquired, they may permit assessment of relative fat or iron content relative to liver, as described for the other AMRI approaches.

#### T2 weighted imaging

T2 weighted imaging is included to increase specificity. Benign lesions like cysts or hemangiomas have high intrinsic T2 signal and can be readily identified, while marked T2 darkness also suggests benignity, which helps with reducing unnecessary call-backs. In contrast, HCC tends to be mildly to moderately T2 hyperintense.

#### Optional: DWI

Similar to NC-AMRI, inclusion of DWI is meant to increase sensitivity for malignancy via a mechanism distinct from HBP imaging. Some DWI features may also be used to help differentiate HCC from non-HCC malignancy, such as intrahepatic cholangiocarcinoma (ICC), as discussed earlier^[43]^.

### Reporting

**Figure 5.**
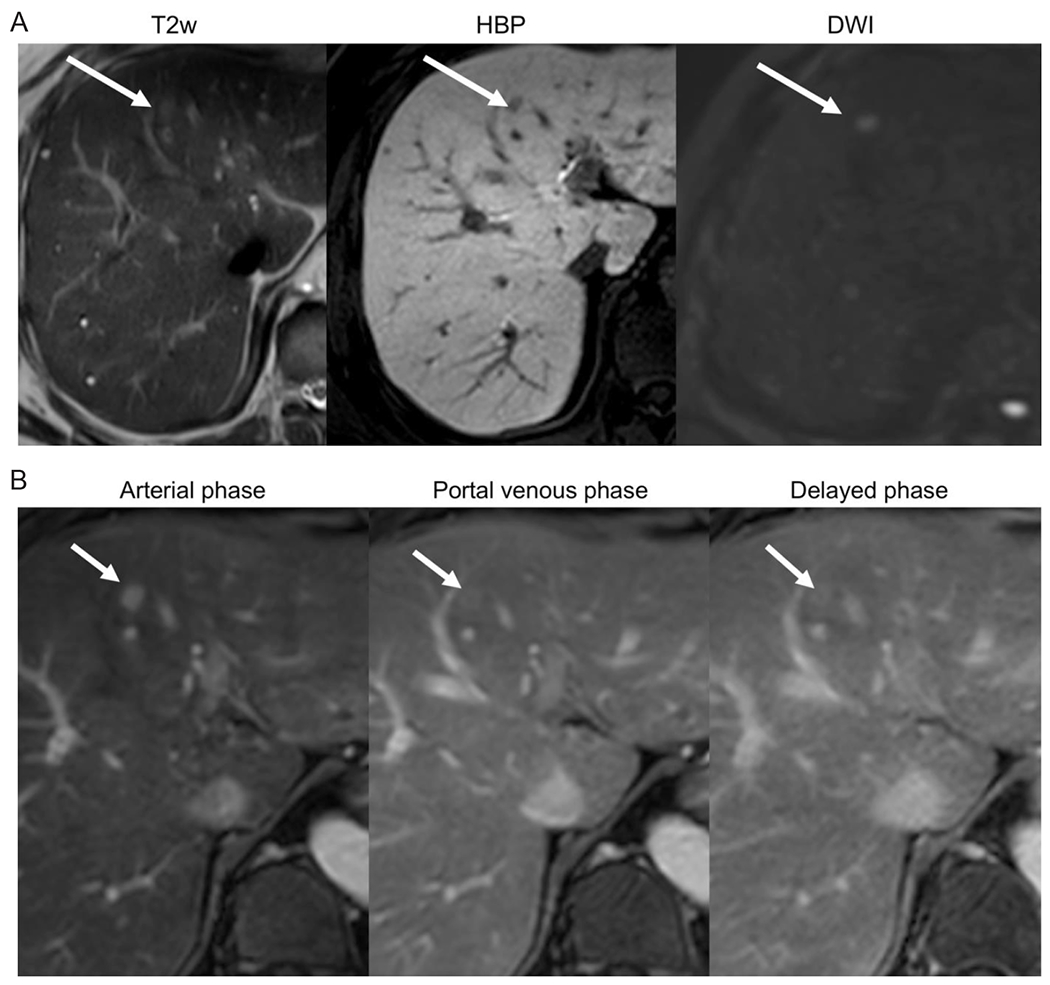
(A) Positive HBP-AMRI examination: 53-year-old male with chronic hepatitis B without cirrhosis. Images show an 8 mm HBP defect in segment 4, with mild T2 hyperintensity and restricted diffusion (arrows). On follow-up extra-cellular contrast MRI dynamic images (B) the lesion exhibits nonrim arterial phase hyperenhancement and capsule. An HCC was confirmed after lesion resection. HBP: hepatobiliary phase; AMRI: abbreviated magnetic resonance imaging; HCC: hepatocellular carcinoma

Reporting of HBP-AMRI is the most developed of all AMRI approaches since HBP-AMRI has been implemented in clinical practice in selected centers in the United States. HBP-AMRI reporting mirrors that of LI-RADS US surveillance reporting with three outcomes: Positive (suspicious nodules ≥ 1 cm), subthreshold (suspicious nodules < 1 cm), and negative (no suspicious nodules)^[49]^. Positive examinations prompt call back for diagnostic MRI or CT. The scoring of HBP-AMRI has been reported previously^[31]^, with an example provided in Figure 5.

### Advantages

HBP-AMRI provides several advantages. The core T1-weighted HBP images have high-contrast-to-noise, aiding in lesion detection. Importantly, hepatocytes retain gadoxetate for an extended period of time. Thus, images can be repeated as necessary. The 20-min delay also allows hand injection of contrast while the patient is in the waiting room, which simplifies workflow, reduces the time the patient is on the MRI table, thus reducing the examination cost, and diminishes the chance of contrast extravasation. This also eliminates the need for a power injector. Finally, HBP-AMRI are reported and interpreted using a simple scoring system modeled from LI-RADS US surveillance^[54]^, which many radiologists are already familiar with, in theory facilitating implementation.

### Disadvantages

The disadvantages of HBP-AMRI center on the contrast agent used and sequelae from cirrhosis. The contrast agent used in HBP-AMRI, gadoxetate, is more expensive than the extracellular agents used for dynamic-AMRI, which may counterbalance some of the cost gains from a simplified workflow. Patients with advanced cirrhosis may have reduced hepatocyte function, which may limit contrast uptake (i.e., reduced liver to lesion contrast), or may have areas of confluent fibrosis, which may reduce the accuracy for HCC detection by obscuring tumors (false negatives) or being mistaken for tumors (false positives). An additional problem is that HBP-AMRI detects HCC based on a very early alteration during hepatocarcinogenesis, namely reduced expression of the OATP transporter, the molecule required for uptake of gadoxetate into hepatocytes^[55]^, which occurs prior to neoangiogenesis^[56]^. This means very early HCC may be detected as hypointense lesions on HBP-AMRI even before they exhibit APHE, making them impossible to definitively characterize as HCC on call back diagnostic imaging^[9,57]^. Centers that elect to apply HBP-AMRI need to be aware of this potential pitfall and understand that HBP-AMRI will detect some patients with HCC precursor nodules prior to overt malignant transformation. Conversely, some reports have shown that occasionally HCCs can be iso- or hyperintense on HBP imaging and may be mistaken for benign lesions^[15,23,40]^.

### Studies to date

Three studies have retrospectively assessed the performance of a simulated HBP-AMRI (derived from a complete MRI with gadoxetate) for HCC detection in patients with cirrhosis or chronic hepatitis B [Table 2], the largest of which was a dual center study in a surveillance population^[28]^. These studies have reported per-patient sensitivities in the range of 80%-83%, per-patient specificities in the range of 93%-96%, and a per-lesion sensitivity of 85%. One study evaluated the performance of HBP-AMRI interpreted prospectively in an HCC surveillance population, demonstrating a sensitivity of 91% and sensitivity of 99%^[31]^. In this study, 20% of patients had Child Pugh B or C cirrhosis with 12 HCC in the cohort. To our knowledge, this study and the previously discussed study evaluating DWI alone^[34]^ are the only two studies to date evaluating the performance of AMRI interpreted prospectively in the clinical setting. Clinical trials are underway^[58]^.

The financial implications of HBP-AMRI have also been studied. By one estimate, HBP-AMRI screening would result in a 30% immediate cost savings relative to complete contrast enhanced-MRI^[29]^. In another estimate, an HCC screening strategy using HBP-AMRI had a favorable incremental cost-effectiveness ratio (ICER) ($3,000) per quality-adjusted life year (QALY) gained compared to US, across a wide range of HCC incidences^59]^.

### Summary statement

HBP-AMRI, perhaps the most well studied of the AMRI approaches, offers a streamlined workflow with simple, established reporting guidelines, the use of high-contrast sequences that can be repeated if needed, and preliminary studies demonstrating its cost effectiveness and diagnostic performance in surveillance populations. The disadvantages are the potential for reduced accuracy in some patients with advanced cirrhosis, the increased cost of the GBCA used for HBP-AMRI compared to dynamic-AMRI, and the possibility of detecting very early HCCs that cannot be confirmed with currently available diagnostic imaging tests.

## CURRENT ISSUES AND GAPS IN KNOWLEDGE

Despite the growing body of literature suggesting AMRI offers superior sensitivity in HCC detection to that reported for surveillance US, there is insufficient evidence to recommend widespread adoption of AMRI by international guidelines. Prospective studies evaluating the performance and cost-effectiveness of AMRI versus US in surveillance populations for detecting HCC and prolonging life will be needed to inform changes to existing guidelines. Although it may take years for that evidence to be generated, AMRI can be of use today. One potential way to integrate AMRI into current practice is to apply it in patients who have severe limitations of their US examinations, such as those with an US LI-RADS visualization score of C^[54]^, or at the discretion of hepatologists, who might be concerned about the reliability of US imaging for patients with markedly heterogeneous liver parenchyma due to underlying cirrhosis or with poor liver visualization due to large body habitus, ascites, or other factors.

Another challenge of implementing AMRI, at least in the United States, is insurance reimbursement. The overarching goal of AMRI is to leverage the high sensitivity of MRI in a cost-effective manner. Moreover, one of the key elements in evaluating or implementing a surveillance program is the overall cost effectiveness of the approach. However, in order to accurately assess the cost-effectiveness of AMRI there must be a billing mechanism that appropriately reflects the reduced scanner time and other health-economic benefits of the shortened protocols. This mechanism currently does not exist in the United States. Objective assessment and wide-spread implementation of AMRI may require the development of new, exam-specific billing codes, like what was done for MR Elastography in 2019. Other countries will likely have to weigh the efficacy, availability, and relative costs to determine the feasibility of AMRI in practice.

While increasing sensitivity by using AMRI addresses one of the problems of surveillance US, it does not solve the problem of poor compliance with surveillance programs^[60]^. The reasons for poor compliance are complicated and not entirely understood. Contributing factors in the United States may include wait times and access to specialists^[60]^. It is not clear if a surveillance modality that requires intravenous contrast and screening like MRI would pose an additional barrier for patient compliance. There is the potential that the higher sensitivity of AMRI would allow for less frequent surveillance, perhaps from twice a year (the current standard) to only once a year, as has been previously proposed^[61]^. However, increasing the surveillance interval remains a theoretical benefit of AMRI and it is unclear if this would improve compliance^[62]^. The impact of AMRI on surveillance compliance should be included in prospective comparative studies.

No study to date has directly compared the different AMRI approaches, and head-to-head studies will be needed to determine the optimal approach. It is possible that no one approach will be best in all patients, and tailored strategies may be needed.

## FUTURE DIRECTION: MEETING CHALLENGES OF MRI WITH NEW TECHNOLOGY

Existing data suggests that AMRI techniques maintain the high sensitivity of complete MRI examinations, however there remains room for improvement and innovation^[63]^. Human and technical factors can contribute to artifacts and undermine image quality, reducing sensitivity for malignancy, especially small lesions. MRI is extremely versatile with many ways to collect data during image acquisition and continuous development of tools for image reconstruction.

**Figure 6.**
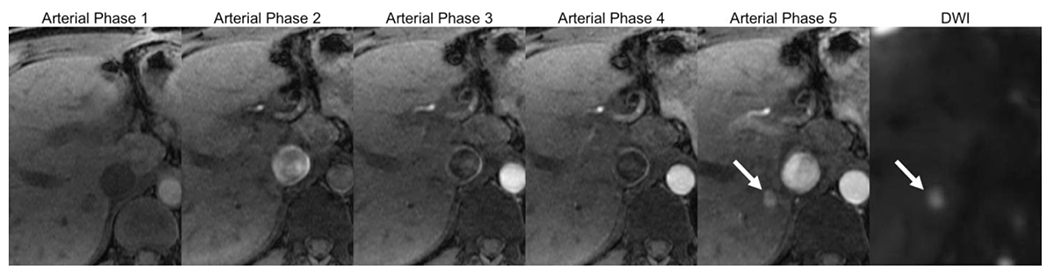
MRI multiarterial phase acquisition (Arterial phase 1-5): the multiphase acquisition in a single breath hold allows capturing the optimally timed arterial phase for HCC detection (in this example, arterial phase 5). A 8 mm observation with nonrim APHE is seen in segment 6, confirmed as a suspicious observation due to restricted diffusion (arrows). MRI: magnetic resonance imaging; HCC: hepatocellular carcinoma; APHE: arterial phase hyperenhancement; DWI: diffusion weighted imaging

Recent advances^[64–70]^ that allow acquisition of multiple arterial phases in a single breath hold are finding their way into clinical practice, increasing the chances of capturing an optimally timed arterial phase, when HCC most commonly shows the highest degree of APHE [Figure 6].

Motion artifacts commonly degrade liver MRI quality. Free-breathing MRI tools are being developed for dynamic post-contrast imaging^[71,72]^, HBP imaging^[73,74]^, and DWI^[75–77]^, as are tools to address cardiac motion, which is particularly problematic in the left lobe of the liver^[78–81]^.

There is great interest in applying artificial intelligence to improve MRI image quality, image registration, and workflow^[73,82–84]^ all of which are active areas of investigation.

## Figures and Tables

**Table 1.** AMRI approaches

	Sequences	Pros	Cons
NC-AMRI	T1 weighted in-phase and out-of-phase T2 weighted imaging Diffusion weighted imaging (DWI)	Cheapest approach Avoids risk of GBCA No issues with contrast timing	Relies on unenhanced imaging Heavily dependent on DWI imaging, which is prone to artifacts in the upper abdomen HCC may not exhibit restricted diffusion
Dynamic-AMRI	Pre-contrast imaging Arterial phase imaging Portal venous phase imaging Delayed phase imaging	Allows definitive diagnosis of HCC Allows diagnosis of tumor in vein Cheaper contrast agent options	Inability to detect ancillary features of HCC Risk of miscategorization of vascular pseudolesions Dependence on contrast timing, thus repeat imaging requires repeat dose of GBCA or repeat exam Requires power injector
HBP-AMRI	Hepatobiliary phase imaging T2 weighted imaging DWI (optional)	High contrast-to-noise Contrast material can be hand injected in waiting room Contrast material is retained in the liver for prolonged duration providing a long imaging window and allowing all sequences to be repeated if necessary Established scoring system based on LI-RADS US	Contrast agent is expensive Lesions may be obscured by severe cirrhosis Can detect very early HCCs that cannot be confirmed with currently available call-back tests

AMRI: Abbreviated magnetic resonance imaging; GBCA: gadolinium-based contrast agent; HCC: hepatocellular carcinoma; LI-RADS: Liver Imaging Reporting and Data System; US: ultrasound; HBP: hepatobiliary phase

**Table 2.** AMRI studies to date

Author	Year	Context of image interpretation	Approach	Country	Target	Design	Intent of source imaging	Liver disease	Reference standard	Sample size	Analysis	Sens.	Spec.	Comments
Kim *et al.*^[23]^	2014	Simulation	NC-AMRI	Korea	Malignancy*	Retros	Diagnosis	Mixed**	Path or FU	128 pts	Per-patient	0.92	0.78	All lesions less than 2cm
Han *et al.*^[24]^	2018	Simulation	NC-AMRI	Korea	HCC	Retros	Diagnosis	Mixed**	Path, cMRI, FU	247 pts	Per-patient	0.84	0.82	
Chan *et al.*^[25]^	2019	Simulation	NC-AMRI	Australia	HCC	Retros	Diagnosis	Cirrhosis	cMRI	44 pts	Per-patient	0.86	0.86	
Sutherland *et al.*^[34]^	2016	Clinical practice	NC-AMRI*	Australia	HCC	Prosp	Surveillance	Mixed**	Path, cMRI, CT	192 pts	Per-patient	0.83	0.98	NC-AMRI was DWI only
McNamara^[36]^	2018	Simulation	NC-AMRI*	USA	HCC	Retros	Surveillance	Mixed**	Explant	37 pts*	Per-lesion*	0.78	0.88	NC-AMRI was DWI only: 17 HCC
Hecht *et al.*^[35]^	2006	Simulation	Dyn-AMRI	USA	HCC	Retros	Diagnosis*	Cirrhosis	Explant	50 pts*	Per-lesion*	0.68	0.66	All scans at 1.5 Tesla; 19 HCC
Khatri *et al.*^[27]^	2020	Simulation	Dyn-AMRI	USA	HCC	Retros	Diagnosis	Cirrhosis	Path, cMRI, FU	100 pts	Per-patient	0.92	0.88	Used coronal T2 as localizing sequence
Marks *et al.*^[28]^	2015	Simulation	HBP-AMRI	USA	HCC	Retros	Surveillance	Cirrhosis or HBV	cMRI or FU	298 pts	Per-patient	0.83	0.93	
Besa *et al.*^[29]^	2017	Simulation	HBP-AMRI	USA	HCC	Retros	Mixed***	Mixed**	Path or cMRI	174 pts	Per-patient	0.80	0.96	
Tillman *et al.*^[30]^	2018	Simulation	HBP-AMRI	USA	HCC	Retros	Surveillance	Cirrhosis or HBV	Path of cMRI	79 pts*	Per-lesion*	0.85	NR	27 HCC
Brunsing *et al.*^[31]^	2019	Clinical practice	HBP-AMRI	USA	HCC	Retros	Surveillance	Cirrhosis or HBV	cMRI or CT	141 pts	Per-patient	0.91	0.99	

* See comments;

** “Mixed” under “Liver Disease” refers to a cohort or population with mixed etiologies of liver disease which is not easily summarized;

*** “Mixed” under “Intent of source imaging” indicates that imaging included in the study could have been done either for the purpose of diagnosis or surveillance.

AMRI: Abbreviated magnetic resonance imaging; cMRI: complete MRI; CT: computed tomography; DWI: diffusion weighted imaging; Dyn-AMRI: Dynamic abbreviated MRI; FU: follow-up; les: lesions; HCC: hepatocellular carcinoma; NC-AMRI: non-contrasted abbreviated MRI; NR: not reported; Path: histopathology; Prosp: prospective study; pts: patients; Retros: retrospective study; Sens: sensitivity; Spec: specificity; USA: United States

## KEY POINTS

There are three variations of AMRI for HCC surveillance (non-contrast, dynamic, and hepatobiliary), each offering unique advantages.

There is a growing body of literature suggesting the sensitivity of AMRI may be higher than US, however existing data does not yet support widespread adoption of AMRI-based HCC surveillance by international guidelines.

Current utilization of AMRI should focus on patients in whom US-based HCC surveillance is compromised.

Clinical trials directly comparing AMRI to US for HCC surveillance in high-risk populations are underway.

Continued evolution of MRI technology is expected to increase the robustness of AMRI for HCC detection.
